# Understanding the delivery of a Canadian-based after-school STEM program: a case study

**DOI:** 10.1186/s40594-017-0083-2

**Published:** 2017-10-02

**Authors:** Eugenia Duodu, Jessica Noble, Yusuf Yusuf, Camilo Garay, Corliss Bean

**Affiliations:** 1 Youth Research and Evaluation eXchange, Toronto, ON Canada; 2 Visions of Science Network for Learning, Toronto, ON Canada

**Keywords:** STEM, Youth, Community, Evaluation, Science

## Abstract

**Background:**

Due to the rising demands for a Canadian workforce with science, technology, engineering, and math (STEM)-related education, there is a need to increase youth engagement in STEM education and programming. Research, however, has shown that youth residing in low-income communities are disproportionately affected by psychosocial barriers, which often inhibit meaningful engagement in STEM programming. Visions of Science Network for Learning (VoSNL) was designed and implemented to address these existing barriers. VoSNL is a charitable organization in Southern Ontario, Canada, that provides weekly community-based STEM programming to low-income and marginalized youth during out-of-school time. VoSNL programming is delivered directly within the community and is free-of-charge for all youth in order to minimize barriers of physical and financial accessibility. The purpose of this report was to provide a detailed description of a core program within VoSNL—Community Science Clubs—and summarize the findings of a process evaluation, specifically the successes and challenges of implementing a community-based, out-of-school STEM program.

**Results:**

Program successes are outlined along with the challenges that have been identified through program implementation. Successes include (a) delivering the program within a community context, (b) opportunities for consistent engagement, and (c) establishing positive youth-staff relationships. Challenges include (a) navigating community-based issues, (b) conducting outreach and promotion, and (c) accommodating a wide age range of youth. Further, lessons learned from an evaluation of program implementation are also discussed.

**Conclusions:**

This report provides one of the first program descriptions and process evaluations of a community-based, youth-focused STEM program within a Canadian context. The findings in this report have helped to improve the delivery and evaluation of the VoSNL program and may act as a catalyst for program expansion to reach more youth in marginalized communities. Further, the findings can also provide a strong framework for programmers interested in implementing STEM youth programming in a community context, assist in the replication of similar models in other locations, and enhance STEM learning amongst youth.

## Findings

There is a growing amount of evidence to support the notion that youth require a strong foundation in science, technology, engineering, and math (STEM) education in order to enhance economic well-being and quality of life during later years in life (Ejiwale, 2013; Let’s Talk Science & Amgen Canada, 2014; President’s Council of Advisors on Science and Technology, 2010). Across North America, the importance of STEM education at the post-secondary level has been emphasized as a significant portion of careers will require some form of STEM literacy and skills (Let’s Talk Science & Amgen Canada, 2014; Mahy & Krimmel, 2008; President’s Council of Advisors on Science and Technology, 2010). In addition to increasing future career options, sustained exposure to STEM learning from kindergarten to grade 12 into post-secondary can foster important critical thinking, problem-solving, and analytical skills (Let’s Talk Science & Amgen Canada, 2014); however, enrolment in STEM-related fields remains low (American Association of State Colleges and Universities, 2005).

Although the significance of STEM learning is well-documented throughout North America (e.g., Afterschool Alliance, 2013; Evans, Lopez, Maddox, Drape & Duke, 2014; Krishnamurthi, Bevan, Rinehart, Coulon, & Ragan, 2013), youth engagement within this domain remains a challenge (Let’s Talk Science and Amgen Canada, 2013; Lyon, Jafri, & St. Louis, 2012). Research has shown that while the majority of Canadian youth recognize the importance of STEM, there is low interest in pursuing STEM learning beyond the compulsory courses at the secondary school level with less than 50% of students, across a selection of provinces, completing grade 11 and 12 math and science courses (Angus Reid Study, 2010; Ipsos Reid, 2010; Lyon, et.al, 2012). This broad disengagement amongst youth has been largely attributed to negative perceptions about science, an inability to perceive connections between STEM education and future career opportunities, and a lack of active and integrated learning approaches (Ejiwale, 2013; Let’s Talk Science & Amgen Canada, 2013, 2014; Lyon, 2010). A recent provincial study by the Higher Education Quality Council of Ontario found that a key determinant of students’ decision to stay on the *STEM pathway* was achievement in science and math courses at the compulsory level (Dooley, Payne, Steffler, & Wagner, 2016). The study found that only 39% of incoming grade 9 students completed grade 12 math and even less (29%) completed grade 12 science (Dooley et al., 2016). While similar studies have not yet been conducted on a national level and key factors related to students’ success are yet to be determined, strategies have been suggested to minimize these identified issues. These include increasing the involvement of youth in STEM learning at an early age, fostering their interest in STEM throughout elementary and high school to ensure a basic level of literacy, and enhancing their awareness of STEM-related opportunities when making decisions regarding future career and educational aspirations (Falk et al., 2016; Nugent, Barker, Welch, Grandgenett, & Nelson, 2015). Several Canadian STEM-based outreach organizations have developed national initiatives to support STEM learning amongst school-aged youth both inside and outside of the school environment, including Actua™ Canada, Let’s Talk Science, and Youth Science Canada.

Despite these efforts to increase STEM engagement amongst youth through targeted programming, there are demographics of youth who are disproportionately underrepresented in STEM fields. These include youth living in low-income communities who are marginalized as a result of their socio-economic status. In major Canadian cities, the majority of youth living in low-income communities are racialized (i.e., visible minority excluding Aboriginal populations), which can often augment their marginalization (Lyon, et al., 2012; National Council of Welfare Reports, 2013). Data from district school boards of major urban cities within Ontario, Canada, indicates a strong relationship between the low socio-economic of youth and their underachievement in STEM-related areas based on standardized tests at the elementary school level (Brown et al., 2015). Further, research has shown that youth from low-income communities can become underrepresented in STEM disciplines within secondary school and future careers as a result of multiple structural and perceived barriers to meaningful engagement at an early age (Grossman & Porche, 2014; Krishnamurthi, Ballard, & Noam, 2014; National Research Council, 2009). These barriers can include a lack of finances for enriched STEM programs and an overall lack of access to and awareness of STEM programs, mentors, and future career opportunities (Grossman & Porche, 2014). Given the potential economic and personal gains of STEM learning, the underachievement and underrepresentation of youth from low-income communities needs to be addressed through facilitating access to meaningful engagement opportunities and reducing existing barriers.

Engaging youth in informal STEM learning environments outside of school time is emerging as an effective strategy in reaching demographics of youth who are commonly underrepresented in STEM learning, including youth from low-income communities (Baran, Bilici, Mesutoglu, & Ocak, 2016; Krishnamurthi et al., 2014; National Research Council, 2009; Vossoughi, 2017). Out-of-school time STEM learning can provide a more contextually relevant, relaxed, and experiential learning environment than those defined by curricula and tests (Blyth & Walker, 2017; Falk, 2017; Noam, Biancarosa & Dechausay, 2003; Nugent et.al, 2015). Activities within these environments are commonly hands-on, student directed, and provide opportunities for collaborative knowledge and skills development (Falk, 2017; Hussar, Schwartz, Boiselle & Noam, 2008; Tan & Barton, 2017). Research has shown the successful delivery of these activities, specifically the recruitment and retaining of program participants, is dependent on the out-of-school component (Vossoughi, 2017). Additionally, out-of-school programming, such as STEM learning, has also shown to produce outcomes in academic achievements, school engagement, and graduation rates (Falk, 2017; Vossoughi, 2017). These non-compulsory STEM-learning environments hold great potential in positively impacting the interest, participation, and the academic achievement of youth (National Research Council, 2008). Thus, such programs can potentially bridge the opportunity gap that low-income youth face by providing enriched engagement and in turn increase the relevance of STEM learning (Afterschool Alliance 2011; Ejiwale, 2013).

Engaging youth from low-income communities in community-based programs requires a more deliberate approach than typical youth STEM programming. Research has shown that youth from low-income families are less likely to participate in community programs that have a fee associated with them or that are located a distance away from their residence (Lopata & Grundmann, 2015; Schnirer et al., 2012). Furthermore, Krishnamurthi (2017) outlined that “after-school [STEM] programs provide an opportunity to engage children from the very populations who are traditionally underrepresented in STEM fields” (p. 3). As such, there is a need for free, accessible, and structured informal STEM-learning programs that are applied and practical, which engage youth from low-income communities. One organization that was developed to address these gaps is Visions of Science Network for Learning (VoSNL) Inc. VoSNL is an organization that was developed to increase access and reduce identified barriers to STEM learning for marginalized youth, specifically with the development of the VoSNL Community Science Clubs (CSC). To date, there is limited research on community-based STEM programs specifically targeting low-income youth within a Canadian context. Several studies have identified a need for improving STEM-based programs (e.g., National Research Council, 2008; National Science Foundation, 2007); however, the majority of studies that have been conducted with out-of-school STEM programs have focused on outcomes rather than processes (e.g., Afterschool Alliance, 2011; Afterschool Alliance, 2013; Krishnamurthi et al., 2014). The dearth of available research focusing on the processes of STEM programming highlights a need to understand the processes in planning and delivering a successful STEM program. As one of the few community-based organizations offering STEM programming in a Canadian context, and to address the aforementioned gaps in the literature, the purpose of this report was to (a) provide a detailed description of the program and (b) present the findings of a process evaluation that outlined the successes and challenges of implementing a community-based STEM program for youth living in low-income, marginalized communities.

### Program description

VoSNL is a charitable organization that aims to advance the educational achievements and career aspirations of youth from low-income and marginalized communities through meaningful engagement in STEM fields and research. Since incorporation in 2004, VoSNL has engaged over 10,000 youth between grades 3 and 8 in STEM activities and maintains a strong commitment to supporting economically disadvantaged youth within several communities within a major city in Ontario, Canada. Based on the researchers’ knowledge, VoSNL is currently the only STEM-based organization in Southern Ontario that delivers programming directly within low-income communities in collaboration with social housing partners. One program stream of this organization is the CSCs.

#### Community Science Clubs

The CSCs were developed to provide hands-on STEM engagement opportunities directly to youth living in low-income communities. The program utilizes a *for the community, in the community* delivery approach that aims to address common accessibility and affordability barriers experienced by youth living in a low-income community. To this end, the CSCs operate directly within recreational and common spaces of target communities during out-of-school time and are free of charge for all youth participants. Through established partnerships between housing providers, post-secondary institutions, and community-based organizations, the CSC program has increased its reach from six communities in Toronto (2011) to 14 communities across the Greater Toronto Hamilton Area (2016). Each CSC engages approximately 15 to 20 local youth, yielding an average of 300 youth engaged in CSC programming annually.

The CSCs include active and integrated learning approaches including workshop sessions, guest speakers, field trips, and an annual showcase. The workshop sessions are delivered to all youth from grades 3 to 8 in the form of weekly, 2-h workshops for 7 months of the year (October to April). Over the course of this 7-month period, approximately 23 workshops are offered in different community locations. Workshop sessions are guided by facilitated modules, which involve active, hands-on STEM experiments and activities. Modules are utilized by VoSNL facilitators and volunteers and are designed to build on many concepts taught within the provincial education system for grades 1 to 8. These modules feature career connections and real-world applications related to society, technology, and the environment and also highlight the work of current scientists who are representative of the diverse backgrounds present within the communities. Aside from workshop modules, the CSCs’ host guest speaker visits from local partner institutions and STEM-based organizations to help build community connections and introduce youth to STEM-related career opportunities. Through various partnerships, CSCs also facilitate active learning opportunities through excursions to various STEM-based learning institutions while ensuring transportation and admission costs are covered. At the end of each program year, youth in all 14 CSCs participate in a culminating activity where they share a STEM-based exposition to stakeholders, community members, and the general public with the aim of raising awareness and generating interest about VoSNL’s CSC to the broader community. Further, the event provides an opportunity for youth to showcase their experiments to the broader community (e.g., family, friends, community members) and provides a leadership opportunity for youth to present and learn from their peers.

The CSCs are delivered with a focus on developing youth-led inquiry, team-building, healthy competition, and project-based learning. Sessions are led by a VoSNL program facilitator and a team of volunteers who are recruited from neighboring post-secondary institutions and within the CSC communities. Prior to joining VoSNL, incoming staff and volunteers undergo screening processes. All staff are required to have a working knowledge of STEM learning and experience in teaching and facilitation techniques and working within target communities and managing teams. Volunteer facilitators are also required to have a working knowledge of STEM learning; therefore, the majority of facilitators have educational backgrounds in STEM disciplines. However, due to the multidisciplinary nature of the work in these communities, some facilitators are also from teaching or social work fields. Facilitators are trained bi-annually on module delivery, facilitation and teaching skills, club management, community outreach, and youth engagement to ensure techniques and quality of program delivery are consistent.

As an extension of engagement, CSC alumni in grades 9 to 12 are provided the opportunity to continue participation as “community ambassadors” where they participate in program facilitation, mentorship, and promotion of CSCs in their respective communities. Overall, the CSC program activities aim to achieve the long-term goal of enhancing youth engagement and academic achievement amongst the target population of youth through five outcomes: (a) increased knowledge and interest in STEM subjects and school, (b) increased school connection and success in STEM, (c) increased self-efficacy and interpersonal and intrapersonal skills, (d) increased understanding of diverse approaches to learning science, and (e) application of transferable knowledge and skills (e.g., problem-solving) to everyday life.

### Methods of data collection

A process evaluation was conducted throughout the implementation of CSCs. The evaluation of the program processes utilized three sources of data: (a) weekly logs completed electronically by the program facilitators from each CSC location (e.g., feedback on workshop modules delivery, overall logistics, and participant observations of session reception); (b) end-of-program feedback forms from the facilitators; and (c) end-of-program feedback forms from volunteer program facilitators.

The weekly logs included 11 questions and were completed at the end of each workshop session. Fourteen program facilitators (100% of all program facilitators) completed this (total of 322 logs) over the course of the 23-workshop sessions. Questions included point-of-service feedback including attendance, activity of focus, feedback on the module, what worked well, and areas for improvement for future sessions. Responses in the weekly feedback tended to be short and abbreviated, taking 5–7 min to complete. The end-of-program feedback form for program facilitators and volunteers and was also completed electronically. The feedback forms for program facilitators were comprised of 19 questions and included demographic information (e.g., age, length of involvement, club location), as well as overarching open-ended questions regarding what worked well and did not work well during program delivery, feedback on program location for the CSC, and suggestions for improvement related to curriculum development, outreach, and volunteers. Finally, space was provided for participants to include additional comments. Responses tended to be more detailed and reflective than with the weekly logs taking program facilitators and volunteers 15–20 min to complete. The feedback forms for volunteers were comprised of 19 questions pertaining to demographic information, perceived participant outcomes, and the overall volunteer experience. Respondents, specifically 13 (93%) program facilitators and 38 (54%) volunteers, were representative of all club locations, with participation from at least one facilitator from each of the 14 clubs.

The data were analyzed using an inductive thematic analysis to help understand program processes (Braun & Clarke, 2006) and identify strategies for improvement. The analysis included coding the data into smaller meaning units and organizing the data into themes and sub-themes (Braun & Clarke, 2006). Identification codes are used for each quotation (WL = weekly logs, PP = post-program, L = leader, V = volunteer). For example PP-L-2016 refers to a program facilitator that completed a post-program feedback form in 2016.

### Process evaluation findings and discussion

This paper presents a summary of the CSC process evaluation findings including successes, challenges, and lessons learned associated with the implementation of the youth-focused CSC program designed to address these barriers for low-income communities within marginalized communities in Ontario, Canada. Some findings build upon existing research while other findings bring forth novel insight. As this is a brief report, a summary of findings is presented; however, the integration of excerpts from the process evaluation data support the sub-themes and allowed for a comprehensive understanding of the processes within the program. As such, some quotations from program stakeholders (i.e., volunteers, program leader) have been included in the following sections. Additionally, the discussion has been integrated into the findings section as well.

### Successes

Four main themes, related to successes of implementation and program delivery of the CSC program in communities emerged during the process evaluation, including: (a) delivering the program within a community context, (b) opportunities for consistent engagement, and (c) establishing positive youth-staff relationships.

#### Delivering the program within a community context

A key component of CSC success in program delivery was the community context engrained within the program. The CSCs are offered directly to youth within their communities in response to evidence that low-income families are less likely to participate in enriched out-of-school time programs due to lack of available and accessible options (Lopata & Grundmann, 2015). Exclusion from these programs can contribute to cyclical and generational poverty, which is especially important in this context given the economic opportunities that STEM learning provides (Ontario Task Group on Access to Recreation for Low-income Families, 2009). Potential opportunities and outcomes of delivering a program in a community context include increased accessibility for participating youth; increased safety as caregivers can walk their children to the program and pick them up; increased participation of community members as there are volunteer opportunities for within the program; and relationship building within the community through convening neighbors and fellow youth, and an enhanced sense of community/feeling of belonging (Lopata & Grundmann, 2015).

The findings of the process evaluation aligned with the literature and revealed the delivery of the CSC program directly within community spaces enhanced accessibility for youth and their caregivers. Program staff acknowledged benefits of direct access to STEM programming within their communities. Specifically, several program leaders outlined: “The area is a good location to have science clubs because it's easy for the kids to get to and the community allows the kids to interact/support each other in and out of club” (PP-L-2016) and “Many of the students live nearby, which makes the building an ideal location as it is easy for them to travel to/from club sessions…it is a good location for [name of club]” (PP-L-2016). Additionally, one program leader stated:

[Name of club] is a great location to have the science club. The area is surrounded by [a] number of buildings for families that might be recent immigrants and/or first generation Canadians, which might lack the guidance/support needed for the children as they go through middle school, high school and transition into post-secondary. [Name of club] serves as a means of introducing the youth to the opportunities within STEM early on. (PP-L-2016)

Finally, one of the program leaders outlined that the CSC location was ideal “because the majority of kids were walking distance from the location” (PP-L-2016).

#### Opportunities for consistent engagement

The CSCs operate consistently on a weekly basis over a 7-month period, delivering a total of 23 workshops at each of the 14 community locations annually. In line with existing research, the consistency of the CSC delivery in communities was identified as being critical for program success. Volunteers described the positive group dynamics of the CSC youth: “I was happy to see that there were students who came in every Saturday without fail, excited to work on science. This was very surprising to me but it definitely motivated me to be more involved” and “[Youth] are committed to attending the club weekly and they constantly stay engaged and ready to have fun” (PP-V-2016). A volunteer also outlined how:

The more children attended, the more they got to know one another. For example, during the air pressure [module]…one student showed other students how to make paper airplanes who were struggling before. I saw cohesion in our group of 20 students. (PP-V-2016)

After the final program event, one leader documented:

Everyone left smiling ear to ear. When our bus returned at the end of the day, after a barrage of hugs, it melted my heart hearing the students trying to calculate the number of weeks until next fall when Science Club is set to start up again. (PP-L-2016)

Research in youth and STEM programming has outlined that programs which engage youth over longer periods of time have a greater impact on positive developmental outcomes than programs shorter in duration (Afterschool Alliance 2011; Catalano, Berglund, Ryan, Lonczak, & Hawkins, 2002). Moreover, sustained engagement can build on initial interest and aid in developing STEM-learning identities of youth inside and outside school (National Research Council, 2008). Consistent programming also allows for deeper connections to be made through building peer-to-peer relationships and receiving mentorship from program leaders (Li & Julian, 2012) with the CSCs. The ability to offer consistent programming also opens up the opportunity to perform broad, longitudinal evaluations.

#### Establishing positive youth-staff relationships

As outlined, program leaders within CSCs are commonly STEM professionals or post-secondary students from local or neighboring post-secondary institutions. Mentorship from the program leaders was recognized as having a favorable influence on youth as one volunteer discussed: “Getting to know the kids better really helped them come out of their shell. They became more confident in taking guesses and trying new things knowing that they were in a very open environment that encouraged sharing new ideas” (PP-V-2016). Additionally, a volunteer reflected on this supportive environment as a predominant success of the program:

A big part of the reason students return every [week] is because they enjoy being there. The informal structure of the club, run by volunteers who genuinely care, creates an atmosphere where the students are free to, essentially, play with scientific concepts. (PP-V-2016)

Literature supports these findings as access to meaningful mentorship opportunities from leaders and peers can play a critical role in supporting STEM learning amongst youth within informal learning environments (National Research Council, 2008). Providing youth with access to engage with STEM mentors and professionals on their own terms, in ways that are not always possible during the school day, can also enhance their sense of belonging in STEM (Krishnamurthi et al., 2014). The positive relationships developed between staff and youth participants in VoSNL were seen as one of the main successes of program delivery.

Fostering youth-staff relationships has shown to be an important aspect of the positive program experience. The fact that program staff and volunteers are often community members from the local community in which the CSC program operates is unique and may enhance connections as youth have the opportunity to connect with their neighbors and fellow community members. Additionally, some of the volunteers are alumni of the VoSNL program (i.e., community ambassadors), providing an opportunity to give back to their community and act as a leader and role model to younger youth. Further, these volunteer alumni tend to be close in age to the youth participants (e.g., 14–16 years old). The close age gap provides opportunities for volunteers to share their knowledge regarding the program, academics, and future careers aspirations with the youth (e.g., how to become a community ambassador, what courses to take in high school), while establishing a relatable connection with the youth participants. Having junior leaders of similar age to youth has been identified as a successful element of program delivery in other youth programs (Shaikh, 2017). Further, research indicates that youth greatly benefit from access to caring non-familial adults which has been identified in general youth programs (Eccles & Gootman, 2002; Roth & Brooks-Gunn, 2003), and specifically STEM programs (Cutucache et al., 2016). Future outcome evaluations will assess whether the community context program delivery approach and the community ambassador program enhance the achievement of VoSNL outcomes in participating youth.

### Challenges

Evaluation of the CSC program through program leader and volunteer feedback revealed three challenges related to program implementation. While many of the program successes reinforced existing best practices within literature, the summary of challenges revealed predominantly novel insights in the potential complexity of program delivery within a community context. These challenges included: (a) navigating community-based issues, (b) conducting outreach and promotion, and (c) accommodating a wide age range of youth.

#### Navigating community-based issues

Delivering programming directly within communities requires collaboration with key stakeholders, including local social housing providers, post-secondary institutions, and community groups in each community. These stakeholders offered support for CSC programming, including access to space, outreach, and logistics support. In order to ensure maximum accessibility to target youth, CSCs were delivered in residential community spaces (i.e., recreation rooms of buildings and townhouse complexes) and, in some cases, neighboring schools and/or community centers. This program-delivery strategy, however, was identified as a challenge on a few occasions throughout program implementation. The findings revealed that compromising situations that occurred in the community during programming months may have inhibited youth involvement in CSC. When certain situations arose, such as imminent violence in the community, the CSC program was impacted as sessions were canceled to ensure the safety of the participants and volunteers. Moreover, any issues that occurred within the building (i.e., maintenance or repairs) directly affected the program. A number of program session cancelations occurred due to these issues, resulting in missed programming for participants and reduced exposure to the content. One leader reflected on the impact on participant attendance: “We’ve been running low on student attendance ever since the construction cancellation” (PP-L-2016).

#### Limited resources

In addition to community situations, limited resources within the community locations were also an identified challenge to program success. Engaging in STEM-based workshops can often require access to specific resources and amenities that are built within the program space, such as internet/technology, a kitchen with running water, and teaching aids (e.g., whiteboards). In some communities, there was an absence of these resources in the space provided by community partners as a result of the infrastructure built within the program space itself. As a result of the limited resources available in some of the space provided, the capacity of the location, the delivery of curriculum content, and, in some cases, the development of certain modules was inhibited. One leader discussed:

I’ve seen the impact of our [program] over the year but [the space] is physically limiting and I was worried for a while that if we had any more registrations I might not be able to accommodate the kids that came in. (PP-L-2016)

Another leader reflected: “Access to water (hot and cold) was an on-going issue throughout the year” (PP-L-2016). Additionally, many of the CSC program spaces were shared with various community groups, which was challenging when coordinating program timing and the security of experiment equipment. A program leader commented on the ongoing issue of equipment security:

Many times, our materials had been used or stolen by other clubs at the location. Specifically, for the year end event we had materials, such as yeast and hydrogen peroxide stolen, specifically bought for the event. It has been an ongoing issue throughout the year (e.g., markers, construction paper, science materials). (PP-L-2016)

#### Conducting outreach and promotion

Outreach, promotion, and scheduling conflicts have been a documented issue for service providers within low-income communities (Schnirer et al., 2012). Conventionally, program outreach and promotion for CSCs has been conducted through posting flyers in community spaces, engaging directly with parents and youth after school, and door-to-door promotion, all of which are proven effective strategies to engage low-income families (Schnirer et al., 2012). Substantial effort was required to engage new populations of young students to join the program, as many youth are initially hesitant to join STEM-based programs. The additional outreach effort required for STEM youth programming is in contrast to other conventional extra-curricular activities for youth, such as music- or sports-based programs because of the natural popularity, sense of engagement, and motivation to engage in this activities (Guèvremont, Findlay, & Kohen, 2008).

Additionally, in a few communities where the CSC program was implemented, there were several out-of-school programs to choose from. A program staff reflected on the impact of these activities: “With the weather warming up, it is difficult to compete against other activities. Many students did not come because of soccer practice and some students who did come, left early for swimming practice” (WF-L-2016). A similar sentiment was echoed at another club: “A couple students had mentioned conflict with timing of Visions of Science due to basketball and soccer practice” (WF-L-2016). This has been identified in other youth program literature as a challenge to reach and engagement (Bean, Kendellen, Halsall, & Forneris, 2014). This finding helped reinforce to program staff that offering the program solely once per week was sufficient.

#### Accommodating a wide age range of youth

The CSCs target youth ages 8 to 14 years old. In some clubs, this age difference poses a challenge to effective teamwork and facilitation. Two volunteer facilitators reported issues related to content delivery and suggested potential solutions: *“*There was a significant range in age groups that made it more difficult for some kids to collaborate” (PP-V-2016) and “Given the age differences of the students, there were some tasks that some really young students could not do…the club would have been more effective in student learning if this group were split into two groups” (PP-V-2016).

The recommendation to separate a large group into smaller groups to optimize program delivery and outcomes has been outlined in other community-based youth programming literature (Bean, Forneris, & Halsall, 2014). Moreover, the engagement of older youth with the workshop material was a challenge as one facilitator noted: “I feel some of the older students are slowly becoming disinterested in participating possibly because of how long they've been attending or because they're no longer being challenged by the content” (PP-V-2016).

An unforeseen age-related challenge that emerged in the CSCs was the need to actively engage youth past the cutoff age as one facilitator mentioned:

[There is a need for] programmatic ways to involve or engage those older students as leaders or as volunteers helping with coordination of the club could be a possible solution. Moreover, as these youth progress into high school and so on, (recognizing the limits of VoSNL) are there any resources, opportunities, programs, community partners etc. we can connect them with to further their interest so as not to leave them hanging? (PP-V-2016)

One approach VoSNL implemented to engage those youth is through opportunities to become community ambassadors and volunteers in the program in which they have aged out of. Similar strategies have been utilized in other youth programming contexts, such as summer camps and youth sport programs (e.g., Bean & Forneris, 2016; Kendellen, Bean, Camiré, & Forneris, 2016). Integrating such an opportunity into the program can help with program sustainability, as well as provide opportunities for mentoring and leadership.

### Next steps, future recommendations, and conclusions

This paper represents an important step in responding to calls for increased evaluation in youth programs (Roth & Brooks-Gunn, 2015) and represents a summary of one of the first process evaluations conducted of a community-based STEM program for youth in a Canadian context. The CSC program provides consistent, hands-on STEM-learning opportunities for youth living in low-income housing during out-of-school time. This program is offered directly within social housing communities in order to increase accessibility. The purpose of this report was to provide a detailed description of the program and a summary of the process evaluation findings.

Ongoing evaluation and reflection facilitated the identification of specific program successes, challenges, and lessons learned that emerged from CSC program delivery. This report addresses gaps in the literature concerning the implementation and evaluation of a STEM-based out-of-school program for youth from low-income communities in a Canadian context and the need for more process evaluations within out-of-school STEM programming. Continuous improvements to the program processes and delivery will enhance the quality of programming and achievement of intended outcomes. As noted above, youth benefit from fostering relationships with caring non-familial adults (Cutucache et al., 2016; Eccles & Gootman, 2002). In support of strengthening this area of success, explicit workshop activities and facilitation techniques related to social-emotional learning will be implemented. A STEM-based example of such activities is Actua™ Canada’s debrief and reflection exercises (Actua™ Canada, 2016).

Another ongoing improvement which VoSNL identified was surrounding their evaluation plan. Research has shown that program evaluation provides opportunities for reflection on past and future programming, helps programmers to better understand and improve service delivery, and showcases effectiveness of a program to key stakeholders and the community (Fitzpatrick, Sanders, & Worthen, 2004). Further, when community organizations partner with research partners, there is the benefit of not only bridging the gap between research and practice (Ferguson, 2005; Yuan et al., 2016), but also allowing for a collaborative learning environment that capitalizes on both partners’ expertise (MacPherson & Hall, 2011).

As such, since the evaluation outlined in the report, VoSNL approached Youth Research and Evaluation eXchange (YouthREX)^1^, housed out of a major university within the Toronto area, with the collaborative goal of building evaluation capacity and developing a more comprehensive evaluation plan to assess both process and outcome indicators. Together, VoSNL and YouthREX have worked to create a logic model to provide a visual representation of VoSNL’s CSC program. Additionally, the organizations collaboratively created an evaluation plan with the goals of better understanding the program experience of youth attending VoSNL, examining potential program outcomes, and identifying strategies for continuous improvement. YouthREX supported VoSNL in developing measurement tools that best meet the intended processes and outcomes of the CSC program. Although VoSNL had existing evaluation protocols in place, programmers saw value in attaining evaluation support from YouthREX to build capacity and expand existing evaluation practices. Moving forward, a mixed-methods approach will be used to assess both processes and outcome indicators, which will enhance data triangulation and strengthen results (Creswell & Plano Clark, 2011). Ultimately, the collaboration between VoSNL and YouthREX has enhanced VoSNL’s evaluation capacity and culture while producing evaluation tools (e.g., pre- and post-program surveys) that will be used for long-term program evaluation and improvement. The evaluation plan and tools are in the midst of being piloted within VoSNL programming.

Although there are several processes of CSC program delivery that have been identified as successful, there remain challenges related to navigating community-based issues, conducting outreach and promotion, and accommodating a wide age range. As previously mentioned, the majority of the community-based issues outlined are external to the program. Considering the nature of the issues, it is imperative to remain committed to the communities served, despite these challenges. Further, as an organization, it is important to recognize and address challenges within program implementation to enhance achievement of intended outcomes and program sustainability (Bean, Kendellen et al., 2014). Therefore, constant communication with stakeholders, collaborators, and community members is integral in mitigating the detrimental impacts of these issues and improving future program delivery (Strengthening Nonprofits, n.d.). Currently, program stakeholders are working together to adapt programming to address the identified challenges and better meet program participants’ needs.

Moreover, the development of workshops to cater to possible resource-related limitations underscores the notion that STEM learning can be adaptable across a range of environments. Additional long-term solutions include working with external partners to introduce necessary resources to community spaces as well as with housing providers to improve infrastructure. In the area of outreach and promotion, future strategies are focused on developing partnerships with other community-based groups. For example, in the summer of 2016, VoSNL partnered with a local community-run sport program to offer STEM-based workshops within their existing basketball camp. Moving forward, similar year-round opportunities and methods will be used to promote the CSCs and engage participants in STEM learning.

Based on the identified successes, VoSNL’s CSCs have expanded and moved towards a sustainable program. Through establishing partnerships between housing providers and community groups, the program has scaled from six communities in 2011 to 18 communities across three different regions within South-Eastern Ontario by 2016. This expansion has demonstrated that the model is viable and scalable across different low-income communities with each presenting its own unique challenges and opportunities. Registration intake data collected from 2013 to present indicates that an average 50% of participants are returning to their CSCs each year (VoSNL, 2016). In many cases, youth participants are the strongest advocates who recommend the program to their peers: “I was recommended by [youth participant]; it’s really fun and I didn’t know it was going to be like this and she told me I should try it out” (personal communication 23 September 2016) and “I would recommend [CSC] to people who don’t understand science as well. If you don’t understand and you ask a volunteer to help you, they will stay by your side throughout the experiment and help you understand it the whole way” (personal communication 23 September 2016). As such, although this research provides insights on STEM programming from an adult perspective, which has been done in other STEM-related research (e.g., Krishnamurthi et al., 2013), future research is needed to understand program processes from the perspective of youth, as their voice is critical in program evaluation (Powers & Tiffany, 2006).

This yearly retention has also facilitated the engagement of participants beyond the projected eighth grade cutoff, where youth would typically age out of the program. Many of these alumni participants opted to continue their involvement by engaging as volunteer facilitators in the CSCs in their respective communities and are now identified as *community ambassadors*. This level of sustained participation further underscores the need and value of consistent programming for youth across communities. Moreover, this level of continued engagement from past participants into a leadership position aids in program sustainability as not only is it cost-effective for a charitable organization to have a large volunteer pool to help facilitate programming, but this also allows for that sense of relatedness discussed previously because of the close age gap between youth and program staff. Further, this addition to programming provides an opportunity for those volunteers to develop leadership skills and contribute to their community by giving back to a program they were involved in, which is the ultimate goal of youth development (Bowers et al., 2010; Lerner et al., 2005). Lastly, accommodating the wide age range in the CSCs has posed a challenge for program facilitators. Therefore, the plan is to develop more age-targeted module modifications and facilitation training which should help mitigate these issues. Moreover, in engaging youth beyond the age cutoff, a new structured out-of-school program for youth ages 14 to 18 will be launched in summer 2017.

Although improvements can be made to best meet the needs of all stakeholders and overcome challenges to ensure program sustainability, findings from this evaluation have aided in the improvement of delivering VoSNL CSC programming. Further, results from this descriptive report may also act as a catalyst for expanding the program to more participants and communities. This report can provide a strong framework for other programmers interested in implementing a community-based STEM program for youth and can assist in the replication of similar models in other cities and enhance STEM learning amongst youth. Finally, although this report outlines important processes, future research is needed to assess the outcomes and impacts of community-based STEM programming on youth within a Canadian context.

## Abbreviations

CSC: Community Science Club
VoSNL: Visons of Science Network for Learning STEM Science, technology, engineering, and mathematics

## Footnotes

1 Launched in 2014 and funded by the Ministry of Children and Youth Services, YouthREX works with Ontario’s youth sector to enhance the accessibility of research evidence and evaluation practices with the aim of improving well-being outcomes for youth.

## References

[CR1] Actua™ Canada. (2016). Actua™. Retrieved on 10 March 2017 from http://actua.ca/en.

[CR2] Afterschool Alliance. (2011). STEM learning in after-school: an analysis of impacts and outcomes. Retrieved from http://www.afterschoolalliance.org/STEM-Afterschool-Outcomes.pdf.

[CR3] Afterschool Alliance. (2013). *Partnerships with STEM-rich institutions. Afterschool alert, issue brief. No. 61*. Washington, DC: Author Retrieved on 20 February 2017 from http://www.afterschoolalliance.org/issue_briefs/issue_STEM_61.pdf.

[CR4] American Association of State Colleges and Universitie. (2005). *Strengthening the science and mathematics pipeline for a better America*. Retrieved from http://www.aascu.org/uploadedFiles/AASCU/Content/Root/PolicyAndAdvocacy/PolicyPublications/STEM%20Pipeline.pdf.

[CR5] Angus Reid. (2010). Vision critical survey, commissioned by Let’s Talk Science and Amgen Canada Inc.

[CR6] Baran E, Bilici SC, Mesutoglu C, Ocak C (2016). Moving STEM beyond schools: students’ perceptions about an out-of-school STEM education program. International Journal of Education in Mathematics, Science and Technology.

[CR7] Bean, C. N., Forneris, T, & Halsall, T. (2014). Girls Just Wanna Have Fun: A processevaluation of a female youth-driven physical activity-based life skills program. *SpringerPlus, 3*, 401-415. doi:10.1186/2193-1801-3-401. 10.1186/2193-1801-3-401 PMC4138317 25143873

[CR8] Bean, C. N., Kendellen, K., Halsall, T., & Forneris, T. (2014). Putting program evaluation intopractice: Enhancing the Girls Just Wanna Have Fun Program. *Evaluation and Program Planning, 49*(2015), 31-40. doi:10.1016/j.evalprogplan.2014.11.007. 10.1016/j.evalprogplan.2014.11.007 25528962

[CR9] Bean, C. N., & Forneris, T. (2016). Exploring stakeholders’ experiences of implementing an ice hockey programme for Inuit youth. *Journal of Sport for Development, 4*(6), 7-20.

[CR10] Blyth D, Walker K, Peppler K (2017). Best practices. SAGE encyclopedia of out-of-school learning.

[CR11] Bowers EP, Li Y, Kiely MK, Brittian A, Lerner JV, Lerner RM (2010). The five Cs model of positive youth development: a longitudinal analysis of confirmatory factor structure and measurement invariance. Journal of Youth and Adolescence.

[CR12] Braun, V., & Clarke, V. (2006). Using thematic analysis in psychology. Qualitative Research in Psychology, *3*(2), 77–101.

[CR13] Brown, R. S., Tam, G., & Marmureanu, C. (2015). Toronto district school board maps representing demographics and achievement by geographic area*.* (research report no. 14/15-11). Toronto: Toronto District School Board.

[CR14] Catalano, R. F., Hawkins, J. D., Berglund, M. L., Pollard, J. A., & Arthur, M. W. (2002). Prevention science and positive youth development: Competitive or cooperative frameworks? *Journal of Adolescent Health, 31,* 230–239. doi:10.1016/S1054-139X(02)00496-2. 10.1016/s1054-139x(02)00496-2 12470920

[CR15] Creswell JW, Plano Clark VL (2011). Designing and conducting mixed methods research.

[CR16] Cutucache CE, Luhr JL, Nelson KL, Grandgennett NF, Parreich WE (2016). NE STEM 4U: an out-of-school time academic program to improve achievement of socioeconomically disadvantaged youth in STEM areas. International Journal of STEM Education.

[CR17] Dooley M, Payne AA, Steffler M, Wagner J (2016). Understanding the STEM path through high school and into university programs.

[CR18] Eccles J, Gootman JA (2002). Community programs to promote youth development.

[CR19] Ejiwale J (2013). Barriers to successful implementation of STEM education. Journal of Education and Learning.

[CR20] Evans MA, Lopez M, Maddox D, Drape T, Duke R (2014). Interest-driven learning among middle school youth in an out-of-school STEM studio. Journal of Science Education and Technology.

[CR21] Falk JH, Peppler K (2017). STEM learning. SAGE encyclopedia of out-of-school learning.

[CR22] Falk JH, Staus N, Dierking LD, Penuel W, Wyld J, Bailey D (2016). Understanding youth STEM interest pathways within a single community: The synergies project. International Journal of Science Education.

[CR23] Ferguson J (2005). Bridging the gap between research and practice. Knowledge Management for Development Journal.

[CR24] Fitzpatrick JL, Sanders JR, Worthen BR (2004). Program evaluation: alternative approaches and practical guidelines.

[CR25] Grossman JM, Porche MV (2014). Perceived gender and racial/ethnic barriers to STEM success. Urban Education.

[CR26] Guèvremont, A., Findlay, L., & Kohen, D. (2008). Organized extracurricular activities of Canadian children and youth. *Health Reports, 19,* 65–69. 18847147

[CR27] Hussar, K., Schwartz, S., Boiselle, E., & Noam, G. (2008). Toward a systematic evidence-base for science in out-of-school time: the role of assessment. Prepared for Noyce Foundation, Education, Afterschool & Resiliency, Harvard University and McLean Hospital. Retrieved from http://www.birds.cornell.edu/citscitoolkit/toolkit/steps/effects/resource-folder/The%20Role%20of%20Assessment.pdf

[CR28] Ipsos Reid. (2010). *Canadian Youth Science Monitor: Prepared for the Canada Foundation for Innovation*. Retrieved from https://www.innovation.ca/sites/default/files/news_items/Jun-7-2010.

[CR29] Kendellen K, Bean C, Camiré M, Forneris T (2016). Facilitators and barriers to leadership development at a Canadian residential summer camp. Journal of Park and Recreation Administration.

[CR30] Krishnamurthi A, Peppler K (2017). After-school STEM programming. SAGE encyclopedia of out-of-school learning.

[CR31] Krishnamurthi A, Ballard M, Noam GG (2014). Examining the impact of afterschool STEM programs.

[CR32] Krishnamurthi A, Bevan B, Rinehart J, Coulon VR (2013). What afterschool STEM does best: how stakeholders describe youth learning outcomes. Afterschool Matters.

[CR33] Lerner RM, Lerner JV, Almerigi JB, Theokas C, Phelps E, Gestsdottir S (2005). Positive youth development, participation in community youth development programs, and community contributions of fifth-grade adolescents findings from the first wave of the 4-H study of positive youth development. The Journal of Early Adolescence.

[CR34] Let’s Talk Science. (2013). *Spotlight on science learning: The high cost of dropping science and math*. Retrieved from https://outreach.letstalkscience.ca/images/Research/2013/SpotlightOnScienceLearning-2013.pdf.

[CR35] Let’s Talk Science & Amgen Canada (2014). Spotlight on science learning—shaping tomorrow’s workforce: what do Canada’s teens think about their futures?.

[CR36] Li J, Julian MM (2012). Developmental relationships as the active ingredient: a unifying working hypothesis of “what works” across intervention settings. American Journal of Orthopsychiatry.

[CR37] Lopata JA, Grundmann C (2015). Toronto after-school programs: what really matters?. Prepared for the City of Toronto.

[CR38] Lyon GH (2010). Project Exploration’s personalized curriculum: fostering access and equity in science out-of-school.

[CR39] Lyon GH, Jafri J, St Louis K (2012). Beyond the pipeline: STEM pathways for youth development. Afterschool Matters.

[CR40] MacPherson, I., & Hall, P. (2011). Community-university research partnership reflections on the Canadian social economy experience. University of Victoria. Retrieved on 9 June 2017 from http://dspace.library.uvic.ca/bitstream/handle/1828/5857/Shirley_Jane_MA_2014.pdf?sequence=1&isAllowed=y.

[CR41] Mahy C, Krimmel T (2008). Knowledge-based economics and education: a grand canyon analogy. College Quarterly.

[CR42] National Council of Welfare Reports (2013). A snapshot of racialized poverty in Canada.

[CR43] National Research Council (2008). Rising above the gathering storm: energizing and employing America for a brighter future.

[CR44] National Research Council (2009). Learning science in informal environments: people, places, and pursuits.

[CR45] National Science Foundation. (2007). *A National action plan for addressing the critical needs of the US science, technology, engineering, and mathematics education system*. Retrieved from https://www.nsf.gov/pubs/2007/nsb07114/nsb07114.pdf.

[CR46] Noam G, Biancarosa G, Dechausay N (2003). Afterschool education: approaches to an emerging field.

[CR47] Nugent G, Barker B, Welch G, Grandgenett N, Wu C, Nelson C (2015). A model of factors contributing to STEM learning and career orientation. International Journal of Science Education.

[CR48] Ontario Task Group on Access to Recreation for Low-income Families (2009). Affordable access to recreation for Ontarians: policy framework—everyone plays.

[CR49] Powers JL, Tiffany JS (2006). Engaging youth in participatory research and evaluation. Journal of Public Health Management and Practice.

[CR50] President’s Council of Advisors on Science and Technology (2010). Report to the president: prepare and inspire: K-12 education in science, technology, engineering, and math (STEM) for America’s future.

[CR51] Roth JL, Brooks-Gunn J (2003). What exactly is a youth development program? Answers from research and practice. Applied Developmental Science.

[CR52] Roth JL, Brooks-Gunn J (2015). Evaluating youth development programs: progress and promise. Applied Developmental Science.

[CR53] Schnirer L, Stack-Cutler H, Community-University Partnership for the Study of Children, Youth, and Families (2012). Recruitment and engagement of low-income populations: service provider and researcher perspectives.

[CR54] Shaikh M (2017). Exploring perspectives of youth leadership in a physical activity-based positive youth development program for at-risk youth (Master’s dissertation).

[CR55] Strengthening Nonprofits. (n.d.). Strengthening nonprofits: a capacity builder’s resource library—partnerships: frameworks for working together. Retrieved from http://www.strengtheningnonprofits.org/resources/guidebooks/Partnerships.pdf

[CR56] Tan E, Barton AC, Peppler K (2017). STEM learning in informal settings. SAGE encyclopedia of out-of-school learning.

[CR57] VoSNL. (2016). VoSNL Annual Report 2015-2016. Retrieved from https://static1.squarespace.com/static/53f40ccce4b08efca8cf581e/t/586d5fc5f5e231abff0fa5bd/1483562952481/VOS_Annual_2015-2016_web_spreads.pdf.

[CR58] Vossoughi S, Peppler K (2017). Access and equity in out-of-school learning. SAGE encyclopedia of out-of-school learning.

[CR59] Yuan NP, Gaines TL, Jones LM, Rodriguez LM, Hamilton N, Kinnish K (2016). Bridging the gap between research and practice by strengthening academic-community partnerships for violence research. Psychology of Violence.

